# Establishment and Application of a Rapid Fluorescence-Based RT-LAMP Assay Targeting the CP Gene for Cherry Virus A Detection

**DOI:** 10.3390/microorganisms14030529

**Published:** 2026-02-25

**Authors:** Liangjie Zhang, Wenrong Xian, Haixia Zhu, Yongqiang Ma

**Affiliations:** 1 Academy of Agriculture and Forestry Sciences, Qinghai University, 251 Ningda Road, Xining 810016, China; 15593854879@163.com (L.Z.); xianwr@sina.com (W.X.); zhuhaixia0101@163.com (H.Z.); 2 Qinghai Key Laboratory of Agricultural Pest Comprehensive Control, Qinghai University, 251 Ningda Road, Xining 810016, China

**Keywords:** reverse transcription loop-mediated isothermal amplification, fluorescence visual detection, Cherry Virus A

## Abstract

In order to establish a rapid and sensitive LAMP visual detection method for Cherry Virus A on-site, this study used the conserved fragment of the CVA coat protein (CP) sequence as a template for primer design. The rapid visual LAMP detection method for Cherry Virus A was successfully established by optimizing the reaction system components (concentration ratio of internal and external primers, and concentrations of loop primers, Bst DNA, Mg^2+^, dNTPs and betaine) and reaction conditions (temperature and time). This method enables specific detection of Cherry Virus A and facilitates visual inspection of crude nucleic acid extracts within 40 min, significantly reducing the diagnostic turnaround time. The limit of detection is 67.54 pg μL^−1^ (cDNA), which is 100 times more sensitive than PCR. Analysis of 70 field sweet cherry samples revealed an RT-LAMP positivity rate of 91.42%, significantly surpassing the 71.42% achieved by RT-PCR. This method is suitable for the rapid on-site detection of Cherry Virus, and can also provide a theoretical reference for the early diagnosis of cherry viral diseases.

## 1. Introduction

Cherry virus A (CVA), a member of the genus *Robigovirus* (family *Betaflexiviridae*), possesses a positive-sense single-stranded RNA genome of approximately 7400 nucleotides (nt) with a 3’ poly(A) tail. The genome comprises two overlapping open reading frames (ORFs):ORF1 encodes a 266 kDa replicase polyprotein, and ORF2 encodes a 52 kDa movement protein (MP) that facilitates viral cell-to-cell transport. CVA was first isolated and characterized in 1995 from sweet cherry samples exhibiting symptoms of little cherry disease in southern Germany [1]. Since its discovery, CVA has been reported worldwide [2,3]. In China, it was first identified in 2009 [4], and is now recognized as one of the most prevalent and widely distributed cherry viruses in the country [5]. A high incidence of CVA has been detected in ‘Hongdeng’ sweet cherry cultivars across Liaoning and Shandong provinces [6]. Subsequent reports from Japan [7] and India [8] with detection rates of 92% and 58%, respectively further confirm its global prevalence and establish CVA as a predominant pathogen of sweet cherry.

CVA exhibits a broad host range within the genus *Prunus*, naturally infecting sweet cherry (*Prunus avium*) and sour cherry (*Prunus cerasus*), and has also been detected in peach (*Prunus persica*), apricot (*Prunus armeniaca*), plum (*Prunus domestica/Prunus salicina*), and Japanese apricot (*Prunus mume*) [9]. A major challenge in its management is its latent nature; infected trees often remain asymptomatic for extended periods, facilitating the undetected spread of the virus through infected grafting and propagation materials [10,11], This poses a substantial threat to orchard health and long-term yield sustainability. Although primarily spread through vegetative means with no known natural vector [12], CVA is frequently detected in mixed infections with other viruses. Its direct contribution to specific disease symptoms remains ambiguous and is often complicated by these co-infections and varietal differences [2], making reliable detection paramount for disease control. Conventional monitoring and detection methods, including enzyme-linked immunosorbent assay (ELISA), dot-blot hybridization, reverse transcription-polymerase chain reaction (RT-PCR), and quantitative PCR (qPCR), face significant limitations in addressing this challenge [13]. ELISA is often compromised by false-negative results due to antibody variability and inhibitors in plant tissues, while RT-PCR and qPCR—though sensitive—are constrained by their dependency on expensive instrumentation, specialized laboratory settings, and time-consuming procedures. These drawbacks limit their practicality for routine or on-site diagnostics, highlighting a clear technological gap: the need for a detection method that is simultaneously sensitive, rapid, cost-effective, and deployable in field conditions.

Loop-mediated isothermal amplification (LAMP), first developed in 2000 [14], offers a compelling solution to this need. This technique employs 4–6 primers targeting 6–8 distinct regions of the target gene. Using Bst DNA polymerase, it achieves highly efficient, strand-displacing DNA amplification under isothermal conditions (60–65 °C) [15,16]. The incorporation of reverse transcriptase enables the direct detection of RNA viruses as reverse transcription LAMP (RT-LAMP) [17]. Amplification can be visually monitored in real time using intercalating dyes (e.g., SYBR Green I, calcein), eliminating the need for post-reaction processing [18,19,20]. Since its inception, LAMP has demonstrated remarkable versatility, having been successfully applied to detect over 100 virus species across 47 gene families [21,22,23]. including significant pathogens such as SARS-CoV-2 [24], *Tomato spotted wilt virus* (TSWV) [25] and *Rehmannia mosaic virus* [26].

Building on this robust foundation and its proven ability to overcome the key limitations of ELISA and PCR-based assays, RT-LAMP presents itself as an ideal platform for developing a next-generation diagnostic tool for CVA. Considering the widespread global distribution of Cherry Virus A (CVA) across major cherry production regions, which severely constrains the healthy development of the cherry industry and the breeding and promotion of improved varieties, there is an urgent need for efficient detection tools. Conventional methods often lack the sensitivity, speed, or practicality required for large-scale, early-stage screening in breeding programs. Therefore, developing a highly sensitive and user-friendly detection method for CVA is of significant importance. Such a method would facilitate virus identification in germplasm resources, screening of disease-resistant parent lines, and production of virus-free planting materials, thereby providing critical technical support for efficient cherry breeding and sustainable production.

## 2. Materials and Methods

### 2.1. Experimental Materials

#### 2.1.1. Biological Materials

All viral samples used in this study, including *Cherry virus A* (CVA), *Prunus necrotic ringspot virus* (PNRSV), *Prune dwarf virus* (PDV), *Cherry rasp leaf virus* (CRLV), *Cherry green ring mottle virus* (CGRMV), and *Little cherry virus 1* (LChV-1), were collected from symptomatic sweet cherry leaves in five counties (districts) of Qinghai Province, China: Ledu District and Minhe County in Haidong City, Chengbei District and Chengzhong District in Xining City, and Guide County in Hainan Prefecture. Viral identities were confirmed by RT-PCR using virus-specific primers listed in Table 1. The target fragments were then amplified, verified by agarose gel electrophoresis, and stored at −80 °C as cDNA templates for future use.

#### 2.1.2. Key Reagents

The Plant Total RNA Extraction Kit (for polysaccharide- and polyphenolic-rich tissues; RNAprep Pure) and the FastKing gDNA Dispelling RT SuperMix were purchased from TIANGEN Biotech (Beijing, China). The Isothermal Amplification 2× LAMP Master Mix and dNTPs (10 mM each) were sourced from Sangon Biotech (Shanghai, China). M-MLV reverse transcriptase (10,000 U/mL), MgSO_4_ (100 mM), betaine, and Bst 2.0 WarmStart^®^ DNA polymerase were obtained from New England Biolabs (NEB, Ipswich, MA, USA). The 1000× SYBR Green I nucleic acid gel stain was purchased from Solarbio Life Sciences (Beijing, China).

#### 2.1.3. Key Equipment

The experiments were conducted using the following instruments: a NanoDrop One Microvolume UV-Vis Spectrophotometer (Thermo Fisher Scientific, Waltham, MA, USA), a 96-Well Thermal Cycler (Applied Biosystems, Carlsbad, CA, USA), a T100 Thermal Cycler (BIO-RAD, Singapore), an Allegra X-64R High-Speed Refrigerated Microcentrifuge (Beckman Coulter, Indianapolis, IN, USA), a ChampGel 6000 Gel Imaging System (Tanon, Shanghai, China), a PRACTUM 213-1CN Analytical Balance (Sartorius, Beijing, China), and a PowerPac™ Universal Electrophoresis Power Supply (BIO-RAD, Hercules, CA, USA; Cat. No. 042BR12905).

### 2.2. Experimental Methods

#### 2.2.1. Primer Design

**Figure 1:**
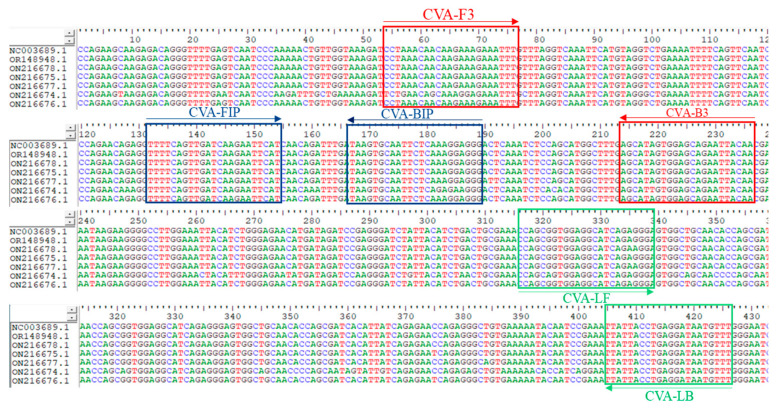
Multiple sequence alignment of the coat protein (CP) gene from various Cherry virus A (CVA) isolates. The red box: the conserved sequence for outer primers.The blue box: the sequence for inner primers. The green box: the sequence for loop primers; indicating a highly conserved and identical DNA fragment across all aligned viral sequences.

The Genomic sequences of CVA were downloaded from GenBank (Accession Numbers: NC_003689, OR148948.1, ON216678.1, ON216675.1, ON216674, ON216676.1 The conserved region of the Coat Protein (CP) gene nucleotide sequence from a GenBank-deposited CVA isolate (Accession Number: ON216677.1) was subjected to multiple sequence alignment analysis using BioEdit software (Figure 1). A relatively conserved low-variability region was selected as the template to design five sets of specific RT-LAMP primers online using PrimerExplorer V5, with RT-PCR primers were designed concurrently (Table 2). Prior to synthesis, the specificity of the primer sequences was verified in silico through BLAST (https://blast.ncbi.nlm.nih.gov/Blast.cgi?CMD=Web&PAGE_TYPE=BlastHome) analysis against non-target virus genomes to minimize the risk of cross-reactivity.

#### 2.2.2. RNA Extraction and cDNA Synthesis

Total RNA was extracted from sweet cherry samples using a Plant Total RNA Extraction Kit designed for polysaccharide- and polyphenolic-rich tissues. The quality of the extracted RNA was verified by 1.5% agarose gel electrophoresis, which revealed intact 28S and 18S rRNA bands, with the 28S rRNA band showing greater intensity than the 18S rRNA band. RNA concentration was quantified at 355.9 ng/μL, with an A_260_/A_280_ ratio of 2.11, as measured using a microvolume nucleic acid quantification system. High-quality total RNA was used for cDNA synthesis using the FastKing gDNA Dispelling RT SuperMix (TIANGEN). The 20 μL reverse transcription reaction consisted of 4 μL of 5× FastKing RT SuperMix, 1 μL of total RNA template (50 ng–2 μg), and RNase-free ddH_2_O added to the final volume. The reaction was performed under the following thermal conditions: incubation at 42 °C for 15 min for reverse transcription, followed by enzyme inactivation at 95 °C for 3 min. The synthesized cDNA products were subsequently verified by PCR amplification of the target gene. Only RNA templates that yielded a clear positive band in the PCR verification were stored at −20 °C for subsequent experiments.

#### 2.2.3. RT-LAMP Primer Screening

To evaluate primer reliability, CVA was first identified and confirmed by PCR amplification with virus-specific primers, followed by agarose gel electrophoresis and sequence alignment. Subsequently, the five designed LAMP primer sets (Table 1) were evaluated using cDNA derived from CVA-positive plants as templates. The 25.0 μL LAMP reaction mixture contained 12.5 μL of Universal 2× LAMP Master Mix, 2.0 μL of CVA-FIP/BIP primers (10 μmol·L^−1^), 0.5 μL of CVA-F3/B3 primers (10 μmol·L^−1^), 1.0 μL of CVA-LB primer (10 μmol·L^−1^), 0.5 μL of DNA polymerase, 1.0 μL of cDNA template (675.4 ng per reaction), and ddH_2_O added to the final volume. Reactions were incubated at 65 °C for 45 min, with healthy tissue-cultured sweet cherry seedlings included as negative controls for each primer set. Following amplification, primer reliability was validated using two parallel detection methods: agarose gel electrophoresis of 2.5 μL of the amplification product to visualize ladder-like banding patterns, and direct in-tube staining by adding 0.5 μL of SYBR Green I to the reaction mixture, followed by brief centrifugation and visual assessment of color change (fluorescent green for positive reactions and orange-yellow for negative reactions).

#### 2.2.4. Optimization of Reaction Components for CVA RT-LAMP Detection

Subsequently, single-factor optimization experiments were conducted to improve the performance of the visual LAMP-based detection system. Key components—including Mg^2+^, dNTPs, Bst 2.0 DNA polymerase, betaine, and primer ratios—were systematically tested based on the initial reaction system (Table 3). The optimal concentration for each variable (inner-to-outer primer ratio, loop primer concentration, Mg^2+^ concentration, dNTPs concentration, Bst 2.0 DNA polymerase concentration, and betaine concentration) was determined by assessing the clarity of ladder-like bands on agarose gel electrophoresis, together with the fluorescence intensity observed after direct addition of SYBR Green I to the reaction tubes post-amplification.

#### 2.2.5. RT-LAMP Temperature Optimization

Based on the optimized RT-LAMP system, temperature optimization was performed across eight gradients (58, 59, 60, 62, 65, 67, 69, and 70 °C) using CVA cDNA as the template. Following amplification, two detection methods were applied: (1) 2.5 μL of the amplification product was subjected to electrophoresis on 1.5% agarose gels, and (2) 0.5 μL of 1000× SYBR Green I was added directly to the reaction tubes for colorimetric assessment. The optimal temperature was ultimately determined based on the sharpness of the ladder-like bands observed on the agarose gel, as well as the brightness of fluorescence resulting from in-tube staining with SYBR Green I in the reaction tubes post-amplification.

#### 2.2.6. RT-LAMP Incubation Time Optimization

Based on the optimized RT-LAMP system, reaction time optimization was performed across six gradients (25, 30, 35, 40, 45, and 50 min) using CVA cDNA as the template. This gradient was designed to systematically determine the incubation time that achieves an optimal balance between assay rapidity and detection sensitivity. Following amplification, 2.5 μL of the products were analyzed by 1.5% agarose gel electrophoresis, and 0.5 μL of 1000× SYBR Green I was added to the reaction tubes for fluorescence visualization. The optimal incubation time was ultimately determined based on a combined assessment of band sharpness on agarose gels and fluorescence brightness after SYBR Green I addition.

#### 2.2.7. RT-LAMP Sensitivity Testing

Using preserved CVA cDNA as the template, concentrations were quantified with a microvolume nucleic acid spectrophotometer, followed by 10-fold serial dilutions (10^0^ to 10^−6^) in DEPC (diethyl pyrocarbonate)-treated ddH_2_O. Both RT-PCR and RT-LAMP were performed with each diluted template. Following amplification, 2.5 μL of the products from each reaction were subjected to 1.5% agarose gel electrophoresis, and the comparative sensitivity of the two methods was evaluated based on electrophoretic band patterns combined with RT-LAMP fluorescence visualization results.

#### 2.2.8. RT-LAMP Specificity Testing

The specificity of the optimized RT-LAMP assay was validated using cDNA samples confirmed positive for six sweet cherry-infecting viruses—CVA, PNRSV, PDV, CRLV, CGRMV, and LChV-1—via previous RT-PCR detection. Primer sequences for PNRSV, PDV, CRLV, CGRMV, and LChV-1 were adopted from [5]. The RT-PCR reactions were performed in a 25.0 μL mixture containing 12.5 μL of 2× Trio Taq Mix, 0.5 μL each of forward and reverse primers, and 1.0 μL of cDNA template (675.4 ng/μL), with nuclease-free ddH_2_O added to the final volume. The thermal cycling conditions consisted of initial denaturation at 95 °C for 5 min, followed by 35 cycles of denaturation at 95 °C for 30 s, annealing at 60 °C for 30 s, and extension at 72 °C for 1–2 min (according to amplicon length), and a final extension at 72 °C for 10 min. PCR products were stored at 4 °C prior to analysis.

Subsequently, cDNA templates from each of the six viruses (in triplicate) were subjected to the optimized RT-LAMP assay. Amplification specificity was assessed in parallel by agarose gel electrophoresis and real-time visual detection. The target band (~202 bp) obtained from the CVA-positive RT-LAMP reaction was gel-purified, cloned, and sequenced. Sequence homology analysis via NCBI BLASTn confirmed the accuracy of the CVA RT-LAMP assay.

#### 2.2.9. Development of a Crude Nucleic Acid-Based RT-LAMP Rapid Detection Technology

Nucleic acids were extracted from plant tissues using a rapid lysis–dilution protocol. Briefly, fresh leaf tissue (20–50 mg) was finely chopped, placed in a 2 mL microcentrifuge tube containing steel beads, flash-frozen in liquid nitrogen, and homogenized by grinding. Subsequently, 500 µL of freshly prepared, pre-chilled lysis buffer (0.1 M sodium citrate [pH 6.4], 0.5% sodium dodecyl sulfate [SDS], and 1% β-mercaptoethanol) was added, followed by thorough vortex mixing. The mixture was subjected to thermal denaturation at 95 °C for 5 min in a water bath, and then equilibrated at room temperature for 2 min. Neutralization was carried out by adding 25 µL of 0.1 M HCl with gentle inversion mixing. After centrifugation at 12,000 rpm for 5 min, 150 µL of the supernatant was collected. Finally, the supernatant was diluted 1–5 fold with nuclease-free DEPC-treated H_2_O to optimize the template concentration for subsequent analyses.

To validate the suitability of this crude extract for direct detection, 2 µL of the diluted supernatant was used as the template in the RT-LAMP reaction system containing reverse transcriptase. The amplification results were compared with those obtained using purified cDNA templates. Both types of templates were tested in triplicate to confirm the feasibility of direct RT-LAMP amplification from crude nucleic acid extracts.

#### 2.2.10. Detection of Field-Collected Samples Using RT-LAMP Technology

To assess field applicability, 70 virus-suspected sweet cherry (*Prunus avium*) leaf samples were comparatively analyzed using both our established CVA-specific RT-LAMP assay and conventional RT-PCR. The RT-LAMP protocol was streamlined into a one-step procedure by pre-incorporating 0.125 μL of M-MLV Reverse Transcriptase (10,000 U/mL) into the reaction mixture.

## 3. Results

### 3.1. Screening and Validation of RT-LAMP Primers for CVA

**Figure 2:**
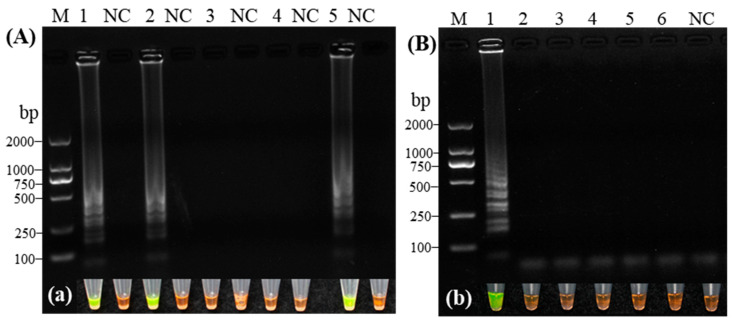
Screening and specificity evaluation of RT-LAMP primer sets for Cherry virus A. Left: Initial screening of five candidate primer sets (Lanes 1–5: Set 1 to Set 5) using CVA cDNA. (**A**) Gel electrophoresis image. (**a**) Corresponding fluorescence visualization. Right: Specificity test of the selected primer set against other cherry viruses (Lanes 1–6: CVA, PDV, PNRSV, CGRMV, CRLV, LChV-1). (**B**) Gel electrophoresis image. (**b**) Corresponding fluorescence visualization; NC, Virus-free plantlets; M, DL2000 DNA Marker.

Amplification was performed using five sets of LAMP primers designed for CVA detection. Among them, primer sets 1, 2, and 5 generated the characteristic ladder-like bands in CVA-positive samples, with no amplification observed in negative controls, confirming the absence of false positives (Figure 2A, left). In contrast, primer sets 3 and 4 produced no amplification products, a result consistent with the corresponding SYBR Green I staining (Figure 2a, left). The specificity of the primers was further validated using cDNA from six sweet cherry-infecting viruses, including CVA, PNRSV, PDV, CRLV, CGRMV, and LChV-1 (Figure 2B, right). Primer set 1 specifically amplified only the CVA template, showing no cross-reactivity with the other five viruses, which was also corroborated by SYBR Green I staining (Figure 2b, right). Based on its reliability and specificity, primer set 1 (containing F3/B3, FIP/BIP, and LB sequences) was selected for all subsequent CVA detection assays.

### 3.2. Analysis of Optimization Results for Component Concentrations in the CVA RT-LAMP Detection System

#### 3.2.1. Determination of Optimal RT-LAMP Reaction Parameters

**Figure 3:**
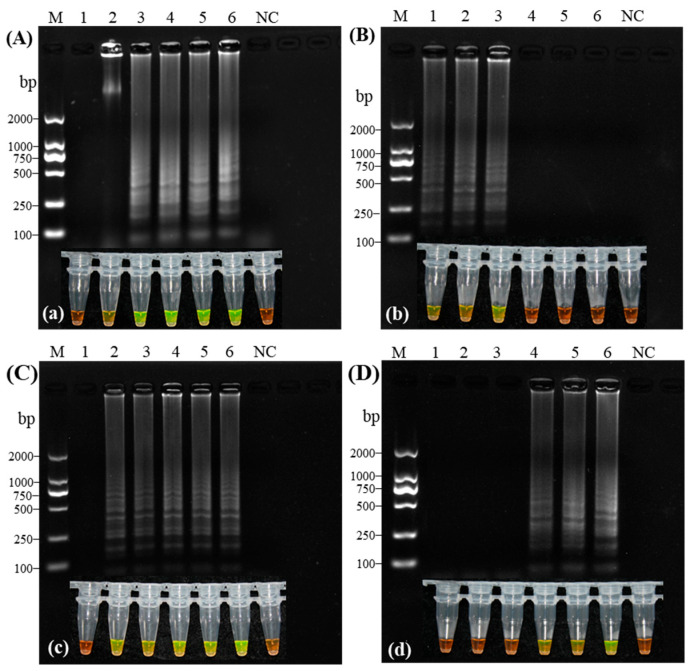
Optimization of key reaction components for the CVA RT-LAMP detection system. (**A**–**D**): Gel electrophoresis; (**a**–**d**): Fluorescence visualization; (**A**) dNTP concentrations (0, 0.5, 1.0, 1.5, 2.0, 2.5 mmol/L); (**B**) Betaine concentrations (0, 0.2, 0.4, 0.6, 0.8, 1.0 mol/L); (**C**) Mg^2+^ concentrations (0, 2, 4, 6, 8, 10 mmol/L); (**D**) Bst 2.0 DNA polymerase concentrations (0.128, 0.192, 0.256, 0.320, 0.384, 0.448 U/μL); NC, Virus-free Plantlets; M, 2000 bp DNA Marker.

The RT-LAMP assay was systematically optimized by testing a series of critical component concentrations. For dNTPs, concentrations ranging from 0.0 to 2.5 mmol/L were evaluated. No amplification occurred below 0.5 mmol/L, whereas distinct ladder-like band patterns were consistently observed between 1.0 and 2.5 mmol/L. Based on detection efficiency and practical application, 1.0 mmol/L dNTPs was selected as optimal (Figure 3A,a). Betaine was subsequently tested from 0.0 to 1.0 mol/L. Amplification with clear ladder patterns occurred at 0.0–0.4 mol/L, with the strongest band intensity at 0.2 mol/L; concentrations ≥0.6 mol/L completely inhibited amplification (Figure 3B,b). Mg^2+^ was evaluated from 0 to 10 mmol/L in 2 mmol/L increments. Amplification occurred from 2 to 10 mmol/L, with optimal band resolution at 6 mmol/L; no product formed in the absence of Mg^2+^ (Figure 3C,c). Finally, Bst 2.0 DNA polymerase was tested from 0.128 to 0.448 U/μL. Clear amplification was observed from 0.320 to 0.448 U/μL, with the sharpest band at 0.320 U/μL (Figure 3D,d). In all experiments, visual fluorescence detection (green-positive tubes) corresponded completely with electrophoretic results. Therefore, the finalized reaction conditions were established as: 1.0 mmol/L dNTPs, 0.2 mol/L betaine, 6 mmol/L Mg^2+^, and 0.320 U/μL Bst 2.0 DNA polymerase.

#### 3.2.2. Primer Concentration Gradient Optimization

**Figure 4:**
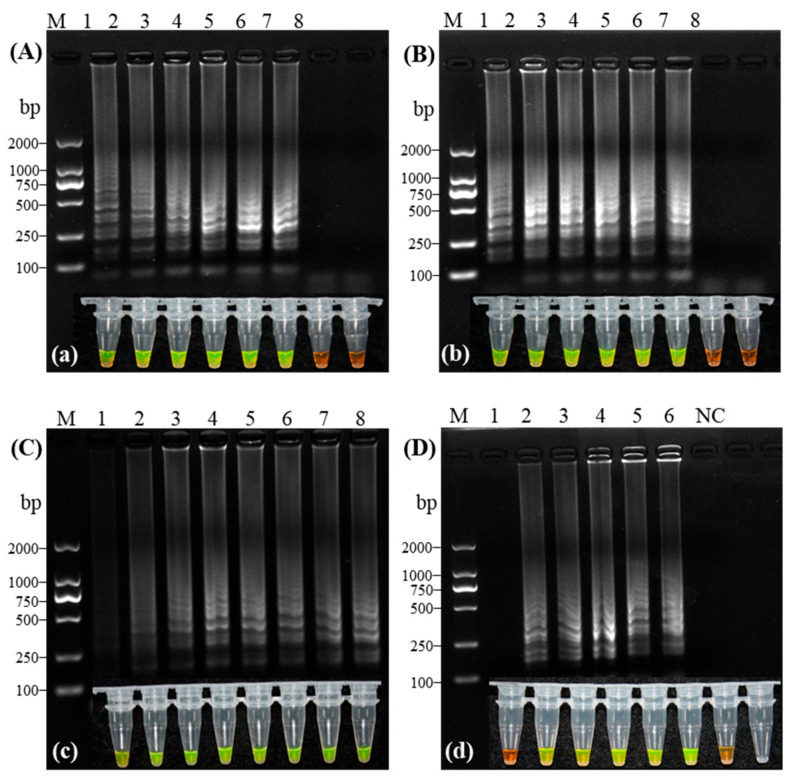
Optimization of primer concentrations, reaction temperature, and time for Cherry virus A RT-LAMP detection. (**A**) Gel electrophoresis analysis of inner/outer primer ratio screening. Lanes 1–6: inner (FIP/BIP) to outer (F3/B3) primer ratios of 1:1, 2:1, 4:1, 6:1, 8:1, and 10:1, respectively, at a fixed outer primer concentration of 10 μmol/L; (**B**) Gel electrophoresis analysis of loop primer concentration screening. Lanes 1–6: loop primer concentrations of 0.0, 0.2, 0.4, 0.6, 0.8, and 1.0 μmol/L, respectively; (**C**) Gel electrophoresis analysis of reaction temperature optimization; Lanes 1–8: reactions performed at 58, 59, 60, 62, 65, 67, 69, and 70 °C, respectively. (**D**) Gel electrophoresis analysis of reaction time-course; Lanes 1–6: amplification products after 25, 30, 35, 40, 45, and 50 min of incubation, respectively. For all panels: lanes marked NC indicate negative control; lane M indicates 2000 bp DNA marker. The initial primer ratio used in (**A**) was 8:1. (**a**–**d**): Fluorescence visualization corresponding to.

Building on the optimized system, both the inner/outer primer ratios and loop primer concentrations were systematically optimized. Primer ratios ranging from 1:1 to 10:1 (six gradients) all produced ladder-like patterns, with the 4:1 ratio yielding optimal band resolution and intensity (Figure 4A). Fluorescence visualization (green-positive tubes) showed complete concordance with the electrophoretic results (Figure 4a). For loop primers (0 to 1.2 μmol/L in 0.2 μmol/L increments), a concentration of 0.4 μmol/L demonstrated the sharpest band definition (Figure 4B), with fluorescence results fully consistent with electrophoresis (Figure 4b). Considering detection efficiency and cost-effectiveness, a 4:1 inner/outer primer ratio and 0.4 μmol/L loop primer concentration were selected for subsequent applications.

#### 3.2.3. Optimization of Reaction Temperature and Time

Based on the optimized system, the reaction temperature was evaluated across eight gradients (58, 59, 60, 62, 65, 67, 69, and 70 °C). Distinct ladder patterns were observed between 60 and 70 °C (Figure 4C), with the sharpest band resolution at 62 °C. All positive reactions within this temperature range exhibited green fluorescence (Figure 4c). Simultaneously, six reaction durations (25, 30, 35, 40, 45, and 50 min) were tested. Amplification occurred between 30 and 50 min (Figure 4D), with the highest band intensity observed at 40 min, which was accompanied by green fluorescence (Figure 4d). Considering the balance between detection efficiency and speed, 62 °C and 40 min were established as the optimal reaction temperature and duration, respectively.

### 3.3. Analysis of Sensitivity Detection Results for RT-PCR and RT-LAMP

**Figure 5:**
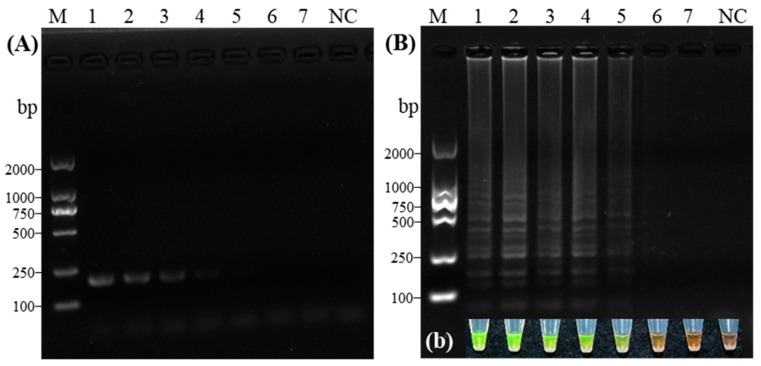
Sensitivity comparison between RT-PCR and RT-LAMP for Cherry virus A detection. (**A**) RT-PCR gel electrophoresis; (**B**) RT-LAMP gel electrophoresis; (**b**) Fluorescence visualization. Lanes 1–7: Serial dilutions of cDNA (675,400 to 0.6754 pg/μL); NC, Negative control; M, 2000 bp DNA Marker.

The cDNA stock solution (675.4 ng/μL) used in this experiment was derived from CVA-positive plant RNA by reverse transcription, followed by PCR amplification and sequence verification. This verified cDNA was then subjected to 10-fold serial dilutions (10^0^ to 10^−6^). Amplification products were analyzed via agarose gel electrophoresis and fluorescence visualization to compare the sensitivity of RT-LAMP and RT-PCR. The results (Figure 5) demonstrated that the RT-PCR detection limit was 6754 pg/μL (Figure 5A), whereas RT-LAMP achieved a detection limit of 67.54 pg/μL (Figure 5B), indicating that the RT-LAMP assay is 100-fold more sensitive than conventional RT-PCR.

### 3.4. Specificity Evaluation of CVA RT-LAMP Detection

**Figure 6:**
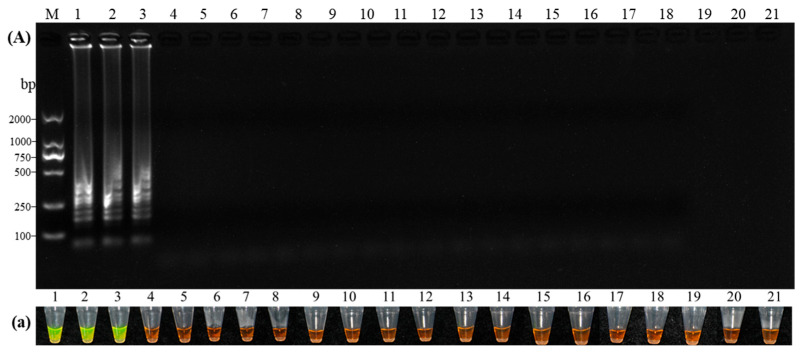
Specificity analysis of the Cherry virus A RT-LAMP detection system. (**A**) RT-LAMP gel electrophoresis; (**a**): RT-LAMP fluorescence visualization. Lanes 1–3: *Cherry virus A* (CVA); 4–6: *Prune dwarf virus* (PDV); 7–9: *Prunus necrotic ringspot virus* (PNRSV); 10–12: *Cherry green ring mottle virus* (CGRMV); 13–15: *Cherry rasp leaf virus* (CRLV); 16–18: *Little cherry virus* 1(LChV-1); 19–20: Virus-free tissue-cultured plantlets; 21: Nuclease-free DEPC-ddH_2_O; M: DL2000 DNA Marker (2000 bp).

The specificity of the optimized RT-LAMP assay was evaluated using cDNA from six prevalent sweet cherry (*Prunus avium*) viruses: CVA, PNRSV, PDV, CRLV, CGRMV, and LChV-1. The results showed that characteristic ladder-like amplification patterns and positive fluorescence signals were generated exclusively in CVA-positive samples. There was complete concordance between electrophoretic and visual readouts, with no cross-reactivity observed for any non-target virus (Figure 6). To further verify amplification accuracy, the target band (~202 bp) corresponding to the CVA amplicon was gel-purified, cloned, and sequenced. Sequence analysis using NCBI BLASTn indicated that the amplified fragment shared 100%, 99.7%, and 99.9% identity with the reported CVA isolates Bd.P12 (GenBank: OR148948.1), PAI3-5-3 (GenBank: ON216678.1), and PVO8-25 (GenBank: ON216675.1), respectively. Together, these results confirm that the established RT-LAMP assay possesses high specificity and accuracy for CVA detection.

### 3.5. Accuracy Validation of Crude Nucleic Acid Extraction for CVA RT-LAMP Detection

**Figure 7:**
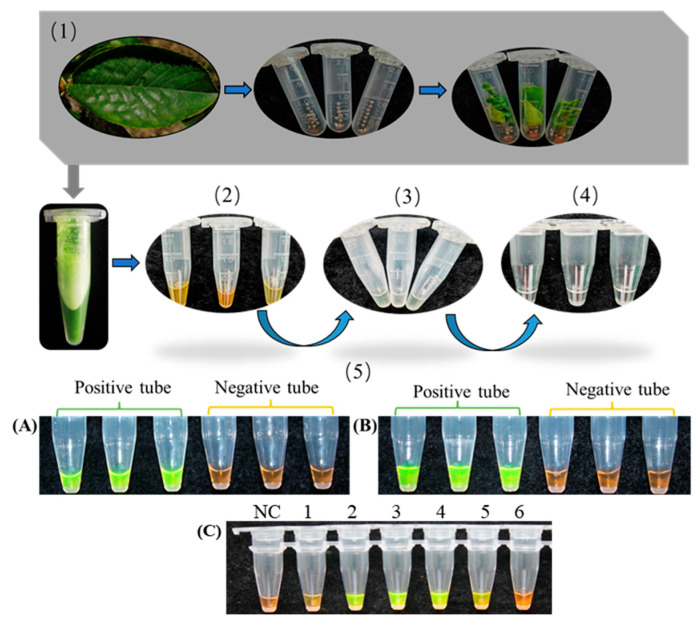
Workflow diagram of crude nucleic acid extraction. (1): Sample Homogenization and Lysis; (2): Neutralization and Clarification; (3): Supernatant Collection; (4): Template Preparation; (5): Visual Analysis: (**A**) Purified cDNA template; (**B**) Crude nucleic acid extract template; (**C**) Fluorescence color development results of LAMP reaction for crude nucleic acid extraction solution; 1–6: LAMP amplification results using 1–6 fold diluted crude nucleic acid extraction solution as template; NC, Negative control.

To streamline plant virus detection, a rapid procedure was adopted: crude nucleic acids were extracted (~15 min) and the resulting supernatant was directly added into the pre-optimized RT-LAMP reaction following reverse transcription (15 min). Amplification was performed for 40 min under closed-tube conditions. Visualization was achieved by pre-depositing 0.5 μL of 10,000× SYBR Green I in the tube caps prior to centrifugation, and the color change was observed immediately after the reaction. As shown in Figure 7, different dilution ratios of the crude extract were evaluated as templates for RT-LAMP. The results indicated that fluorescence visualization was optimal when the crude nucleic acid extract was diluted 2- to 4-fold (Figure 7C). At lower dilution factors (e.g., undiluted or concentrated extract), color development was inconsistent, whereas dilutions beyond 6-fold failed to produce detectable signals. In this study, the total RNA concentration of the crude extracts from all tested samples ranged approximately from 150 to 850 ng/μL, with A_260_/A_280_ ratios between 1.8 and 2.1, indicating the presence of impurities such as proteins but confirming that the RNA content was sufficient for subsequent RT-LAMP detection. Consequently, a 2- to 4-fold dilution range was determined to be optimal for reliable detection.

Validation experiments confirmed that detection results from crude RNA extracts of CVA-infected sweet cherry samples were consistent with those obtained using purified cDNA templates. Positive samples exhibited clear green fluorescence, whereas negative controls remained orange. This integrated approach—combining a 15-min nucleic acid extraction with the optimized RT-LAMP protocol—demonstrates that the assay meets the requirements for rapid, field-deployable diagnostics and confirms its practical applicability.

### 3.6. Field Validation of CVA RT-LAMP Detection

Seventy field-collected sweet cherry leaf samples were concurrently analyzed using both conventional RT-PCR and the established RT-LAMP assay. The results (Table 4) demonstrated that RT-LAMP detected 64 positives (91.42%), significantly surpassing the RT-PCR detection rate of 50 positives (71.42%) (χ^2^ = 9.32, *p* = 0.002). This ~20% increase in detection capacity confirms the superior diagnostic accuracy of the RT-LAMP assay for field applications.

## 4. Discussion

This study presents the first visual loop-mediated isothermal amplification (LAMP) assay suitable for the detection of Cherry virus A (CVA). By targeting highly conserved regions of the coat protein (CP) gene, an optimal primer set (CVA-1) was identified, enabling the development of a streamlined 40-min protocol at 62 °C. The assay demonstrated a 100-fold higher analytical sensitivity than conventional RT-PCR, achieving a detection limit of 67.54 pg/μL, and exhibited absolute specificity with no cross-reactivity against five other prevalent cherry viruses. This level of performance is crucial for identifying latent, low-titer infections that drive the asymptomatic spread of CVA, directly addressing a key diagnostic challenge in its management.

The exceptional performance of the assay stems from a combined strategy of precise primer design and systematic reaction optimization. Primer design followed stringent criteria, including multiple sequence alignment to target conserved regions and in silico validation to minimize primer-dimer formation and off-target amplification, thereby reducing the potential for false-positive results [27,28]. The incorporation of a loop primer (CVA-LB) further accelerated amplification kinetics, enabling detectable results within 30 min and optimal output at 40 min. Systematic optimization of the reaction system revealed that concentrations of Bst 2.0 DNA polymerase, Mg^2+^, and dNTPs were the most critical factors influencing amplification efficiency, whereas temperature, betaine concentration, and primer ratios had a comparatively minor effect. These findings are consistent with optimization patterns reported for other plant virus RT-LAMP assays, such as that for potato leafroll virus [29,30], The final optimized condition of 62 °C for 40 min represents an optimal balance between speed and robustness, collectively establishing a robust and efficient detection system.

The developed assay demonstrates clear practical advantages over existing methods. Compared to a reported RT-LAMP protocol for Tomato spotted wilt virus (TSWV) requiring 40 min at 60 °C [25], our method achieves a 33% reduction in processing time while maintaining high diagnostic reliability. The performance benchmark of this assay aligns with the established capabilities of LAMP technology in plant virology, which is renowned for its high amplification efficiency—often 10- to 1000-fold greater than conventional PCR—making it particularly suited for detecting low viral loads [31]. The achieved detection limit is consistent with other robust LAMP assays, such as those for *Tobacco mosaic virus* and *Yam latent virus* [32], underscoring the general reliability of the technique for trace-level pathogen detection. Field validation confirmed its superior detection rate compared to RT-PCR, largely attributable to its higher sensitivity in identifying samples with low viral loads. This finding is consistent with the conclusions reported by X. Kong et al. [33] in their study on grape downy mildew detection, where the LAMP detection rate (65.2%) was significantly higher than that of PCR (22.2%).

For field deployment, the assay incorporates key features for practicality: the rapid nucleic acid extraction protocol, a visual colorimetric readout that eliminates the need for specialized instrumentation, and a one-step setup using pre-mixed reagents with pre-deposited dye to minimize contamination risk. These attributes render it ideally suited for resource-limited settings such as orchards, nurseries, or ports of entry, enabling rapid on-site screening and timely phytosanitary decision-making.

Nevertheless, certain limitations should be acknowledged. First, although the simplified crude extraction method improves field applicability, the assay’s sensitivity may still be affected by inhibitors present in complex plant tissues. Moreover, the extremely high amplicon yield characteristic of LAMP (10^9^–10^10^ copies/μL) increases the risk of aerosol contamination and potential false positives in open-tube visual detection formats [34,35]. To address these issues and further improve field utility, future efforts could focus on developing lyophilized all-in-one reagents and integrating closed-tube detection systems, such as lateral flow strips. Second, and more importantly, validation based on crude extracts is inherently limited in its capacity to definitively establish the assay’s limit of detection (LoD) or its absolute robustness under field conditions, due to the unknown and variable concentrations of target viral RNA in such samples. Although our results confirm the assay’s ability to detect the virus in complex matrices, a conclusive assessment of its sensitivity and reliability requires comparison against standardized references with known viral RNA copy numbers. Thus, future validation using synthetic RNA controls is essential to determine the exact LoD and to unequivocally verify assay performance under controlled conditions. Finally, this study employed endpoint detection for RT-LAMP, which is suitable for qualitative diagnosis but does not enable real-time quantification of viral load—a feature inherent to qPCR. Subsequent research could explore adapting the assay to a quantitative LAMP (qLAMP) format to permit viral load monitoring. It should also be noted that while discrepancies exist between the RT-LAMP and PCR results, the observed higher detection rate of our RT-LAMP assay aligns with established trends favoring isothermal amplification in field diagnostics. Future studies incorporating sequencing of discordant samples will be crucial to definitively resolve these differences and to confirm viral identity. Additionally, validation across a broader range of *Prunus* species and geog.

## 5. Conclusions

This study developed and validated a novel, rapid, and visual RT-LAMP assay for the detection of Cherry virus A (CVA). The assay exhibits exceptional sensitivity (100-fold higher than RT-PCR), absolute specificity, a short turnaround time (40 min), and instrument-free visual interpretation. These attributes directly address the key diagnostic shortcomings for CVA, providing a practical and powerful tool for on-site diagnosis, nursery certification, and large-scale phytosanitary surveillance. The implementation of this assay is expected to significantly enhance early disease detection and containment efforts, thereby supporting the sustainable production of sweet cherry worldwide.

## Figures and Tables

**Table 1:** Virus specific detection primers used in RT-PCR assay.

Primer	Sequence (5’-3’)	GenBank Accession Number	Location	Product Size/bp	References
PDV-F	CGAAGTCTATTTCCGAGTGG	NC-008038	1340~1359	304	[5]
PDV-R	CCATCGGCTTGTTTCGCTGT		1624~1643		
PNRSV-F	AACTGCAGATGGTTTGCCGAATTTGCAA	FR773524.2	10~128	675	
PNRSV-R	GCTCTAGACTAGATCTCAAGCAGGTC		658~675		
CGRMV-F	TGCGGGAAATCAACTCTTGTC	AJ291761	6276~6296	363	
CGRMV-R	TGTGCCACCAAACACCTTAC		6619~6638		
CRLV-F	TGACTTTCCCAAGGATGAGA	AY964390.2	5311~5330	447	
CRLV-R	GTGACATACCATAGATCC		5740~5757		
LChV-1-F	GGTTGTCCTCGGTTGATTAC	Y10237.1	7090~7109	300	
LChV-1-R	GGCTTGGTTCCATACACTTC		7370~7389		
CVA-F	GCAATTCTCAAAGGAGGGACTCAAATC	PX363516.1	102~209	201	Our laborator
CVA-R	TCTCTGATAATGTGATCGCTGGTGTTG		325~389		

**Table 2:** Oligonucleotide primers targeting the coat protein (CP) gene for detecting Cherry virus A via RT-LAMP and RT-PCR.

Primer Name	Primer Sequence (5′-3′)	Application
CVA-1-F3	CTCCAGCATGGCTTTGAG	The first group of RT-LAMP
CVA-1-B3	TTGTATTTTTCACAGCCCTC	
CVA-1-FIP	CGGATCTATCATGTTCTCCCAGATGATAGTGGAGCAGAATTACAACG	
CVA-1-BIP	ATCTGACTGCGAAACCAGCG GGTTCTCTGATAATGTGATCG	
CVA-1-LB	GGAGTGGCTGCAACACCAG	
CVA-2-F3	GAGGGACTCAAATCTCCAG	The second group of RT- LAMP
CVA-2-B3	TTTTCACAGCCCTCTGGT	
CVA-2-FIP	CCAGATGTAATTTCCAAGGCCC ATGGCTTTGAGCATAGTGG	
CVA-2-BIP	GATCCGAGGGATCTATTACATCTGA ATGTGATCGCTGGTGTTG	
CVA-2-LB	TGGAGGCATCAGAGGGAGTG	
CVA-3-F3	GGTAAAGATCCTAAACAACAAGAA	The third group of RT-LAMP
CVA-3-B3	ATTTCCAAGGCCCCTTCT	
CVA-3-FIP	CAACTGAAAACCTCTGTTCTGGATT TTTGTTTAGGTCAAATTCATGTAGG	
CVA-3-BIP	GATAAGTGCAATTCTCAAAGGAGGG TCTGCTCCACTATGCTCAA	
CVA-4-F3	GGTCAAATTCATGTAGGTCTGA	The fourth group of RT-LAMP
CVA-4-B3	CCTCGGATCTATCATGTTCT	
CVA-4-FIP	CCTCCTTTGAGAATTGCACTTATCA AAATTTTCAGTTCAATCCAGAAC	
CVA-4-BIP	AATCTCCAGCATGGCTTTGAG CCCAGATGTAATTTCCAAGG	
CVA-5-F3	GATTTGATAAGTGCAATTCTCAA	The fifth group of RT-LAMP
CVA-5-B3	GCCCTCTGGTTCTCTGAT	
CVA-5-FIP	GCCCCTTCTTATTTCGTTGTAATTC GGAGGGACTCAAATCTCC	
CVA-5-BIP	GATCCGAGGGATCTATTACATCTGA ATGTGATCGCTGGTGTTG	
CVA-5-LF	CCACTATGCTCAAAGCCATGCT	
CVA-5-LB	AAACCAGCGGTGGAGGCA	

**Table 3:** Optimization gradients for key components in the fluorescence visualization LAMP detection system.

Gradient Parameters	Inner/Outer Primer Ratio *	Loop Primer Concentration (μmol/L^−1^)	Mg^2+^ Concentration (mmol/L^−1^)	dNTPs Concentration (mmol/L^−1^)	Bst 2.0 Polymerase (U·μL^−1^)	Betaine Concentration (mol/L^−1^)
1	1:1	0	0	0	0.128	0
2	2:1	0.4	2	0.5	0.192	0.2
3	4:1	0.6	4	1.0	0.256	0.4
4	6:1	0.8	6	1.5	0.320	0.6
5	8:1	1.0	8	2.0	0.384	0.8
6	10:1	1.2	10	2.5	0.448	1.0

* With the addition volume of outer primers F3/B3 (10 μmol/L) fixed, the initial reaction concentration ratio of inner primers (FIP/BIP) to outer primers (F3/B3, 10 μmol/L) was set at 8:1.

**Table 4:** Field sample detection: RT-PCR vs. RT-LAMP in sweet cherry.

Detection Method	Tested	Positive	Positivity Rate (%)
RT-LAMP	70	64	91.42%
RT-RCR		50	71.42%

## Data Availability

The primers and corresponding gene sequences used in this study have been stored in the NCBI GeneBank database with entry numbers PX363516.1 https://www.ncbi.nlm.nih.gov/search/all/?term=PX363516.1 (accessed on 8 January 2025). In addition, all data generated or analyzed in this study have been incorporated into published articles.
